# Blood-sampling collection prior to surgery may have a significant influence upon biomarker concentrations measured

**DOI:** 10.1186/s12014-015-9093-6

**Published:** 2015-07-31

**Authors:** Nicolas Kahn, Julia Riedlinger, Markus Roeßler, Christina Rabe, Michael Lindner, Ina Koch, Sabine Schott-Hildebrand, Felix J Herth, Marc A Schneider, Michael Meister, Thomas R Muley

**Affiliations:** Department of Pneumology and Critical Care Medicine, Thoraxklinik, University of Heidelberg, Heidelberg, Germany; Translational Research Unit (STF), Thoraxklinik, University of Heidelberg, Amalienstr. 5, 69126 Heidelberg, Germany; Roche Diagnostics GmbH, 68305 Mannheim, Germany; Translational Lung Research Centre Heidelberg (TLRC-H), German Centre for Lung Research (DZL), Heidelberg, Germany; Center of Thoracic Surgery, Asklepios Fachkliniken München-Gauting, Ludwig Maximilians University, 82131 Gauting, Germany; Comprehensive Pneumology Centre Munich (CPC-M), German Centre for Lung Research (DZL), Munich, Germany

**Keywords:** Biomarker, Blood specimens, Sampling time points, Lung cancer, SCC, CEA, CYFRA 21-1, Anesthesia induction, SPREC coding

## Abstract

**Background:**

Biomarkers can be subtle tools to aid the diagnosis, prognosis and monitoring of therapy and disease progression. The validation of biomarkers is a cumbersome process involving many steps. Serum samples from lung cancer patients were collected in the framework of a larger study for evaluation of biomarkers for early detection of lung cancer. The analysis of biomarker levels measured revealed a noticeable difference in certain biomarker values that exhibited a dependence of the time point and setting of the sampling. Biomarker concentrations differed significantly if taken before or after the induction of anesthesia and if sampled via venipuncture or arterial catheter.

**Methods:**

To investigate this observation, blood samples from 13 patients were drawn 1–2 days prior to surgery (T1), on the same day by venipuncture (T2) and after induction of anesthesia via arterial catheter (T3). The biomarkers Squamous Cell Carcinoma antigen (CanAG SCC EIA, Fujirebio Diagnostics, Malvern, USA), Carcinoembrionic Antigen (CEA), and CYFRA 21-1 (Roche Diagnostics GmbH, Mannheim, Germany) were analyzed.

**Results:**

SCC showed a very strong effect in relation to the sampling time and procedure. While the first two points in time (T1; T2) were highly comparable (median fold-change: 0.84; p = 0.7354; correlation ρ = 0.883), patients showed a significant increase (median fold-change: 4.96; p = 0.0017; correlation ρ = -0.036) in concentration when comparing T1 with the sample time subsequent to anesthesia induction (T3). A much weaker increase was found for CYFRA 21-1 at T3 (median fold-change: 1.40; p = 0.0479). The concentration of CEA showed a very small, but systematic decrease (median fold-change: 0.72; p = 0.0039).

**Conclusions:**

In this study we show the unexpectedly marked influence of blood withdrawal timing (before vs. after anesthesia) and procedure (venous versus arterial vessel puncture) has on the concentration of the protein biomarker SCC and to a less extent upon CYFRA21-1. The potential causes for these effects remain to be elucidated in subsequent studies, however these findings highlight the importance of a standardized, controlled blood collection protocol for biomarker detection.

## Background

Lung cancer is still the leading cause for cancer related death in Europe and North America. Despite advances in treatment only 5–15% of patients survive the first 5 years after diagnosis, mainly in function of the initial stage of the disease [1]. In the era of molecular targeted therapies, specific treatments for lung cancer become increasingly important and thus makes molecular biomarker discovery more meaningful for lung cancer management [2, 3].

Molecular biomarkers are developed to assist in the diagnosis, prognosis and treatment monitoring of diseases. The range of potential marker candidates covers the wide spectrum of DNA, RNA, proteins, and other molecules, which may be derived from all kind of body fluids, tissues or even exhaled air (the methylation and mutation detection, sequencing, microarrays, and enzymatic reactions to name only a few). This in turn has driven the development of a number of dedicated diagnostic platforms to aid the detection and measurement of a new generation of advanced biomarkers.

Major efforts are underway to search and validate new biomarkers and extend the range of applications of already known biomarkers, as well as fine tune the methods used to minimize intra- and inter-laboratory variations. Besides technical aspects, biological variations such as diurnal, intra-individual, dietary or long term like in potential aging biomarkers, are known and accounted for [4].

In this study we focused on variations of well-known markers SCC, CYFRA 21-1 and CEA as an effect of anesthesia induction, sampling procedure or time of sampling. These tumor markers are rarely used for the diagnosis of lung cancer due to their lack of sensitivity and specificity. However, the utility of these markers in disease monitoring and prognosis is well established [5–15].

The sampling/collection of blood, tissue, or other bio-specimens required for biomarker determination and general diagnostic purposes mostly follows practical clinical workflow considerations. Efforts are generally made to standardize blood processing and storage conditions [16] but aspects prior to the sample collection procedure are very variable and are difficult to control. For example the timing of blood collection may significantly differ in a hospital versus an outpatient setting. The time point for in-patients is often coupled to diagnostic or treatment procedures (for example endoscopy, surgery, and anesthesia). Apart from these known sources for variation, we wish to illustrate certain under-estimated and frequently unrecognized sources of variability.

The conditions under which the blood samples from study participants are collected are usually inadequately annotated in many cases and researchers need to be alerted to possible biases caused in assessing new serum/blood derived biomarkers. In addition, there is a paucity of available literature focusing specifically on pre-analytical research like potential variations in biomarker levels before or after specific diagnostic or therapeutic procedures or depending on blood withdrawal procedure.

In order to analyze potential effects of blood sampling conditions (i.e. time point: pre-versus post-anesthesia; venous versus arterial) on biomarker concentrations we selected three well-established markers (i.e. SCC, CEA, and CYFRA 21-1) and measured them in a highly annotated fresh cohort of patients.

## Results

**Fig. 1:**
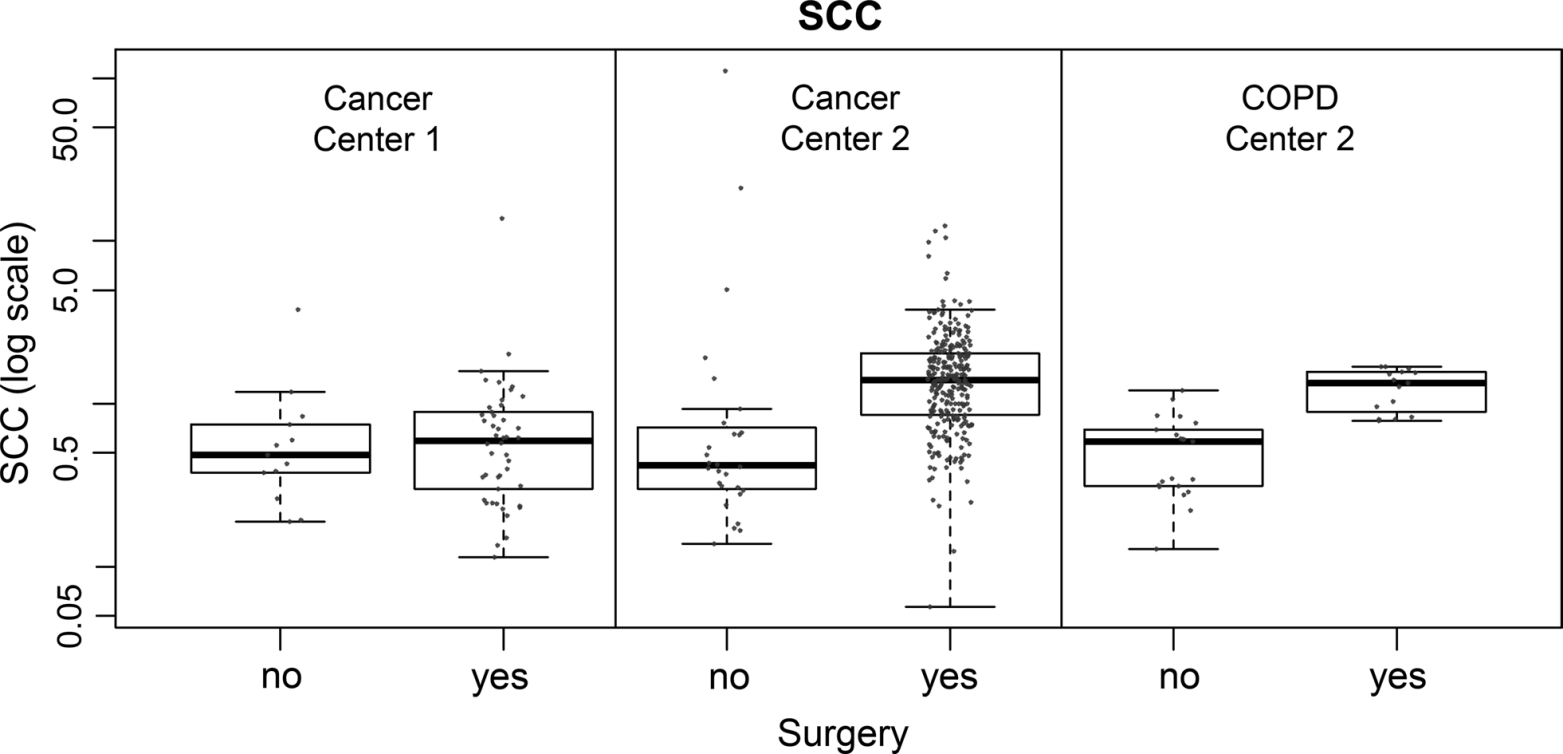
Results of SCC measurement in two centers in respect to whether the patient underwent surgery (OP) or not (no OP). There is an increase in SCC concentrations in center 2 that can be attributed to the presences or absence of surgery independent from common clinical variables like gender, stage, age and histology.

Serum samples from lung cancer patients of 2 lung cancer centers, collected within a framework of a larger Lung Cancer Screening project were compared. The biostatistical analysis of several biomarker candidates revealed unexpected differences between marker values in lung cancer patients depending upon the center where blood sampling was performed. The main source of variation in marker concentration across both centers could be attributed to the fact whether a patient underwent surgery or not. This effect was also visible in surgical patients with COPD. In Fig. 1 an example is given for SCC. The effect was evident especially for samples withdrawn from patients in center 2. After reviewing the process of blood sampling in more detail we established that blood samples of patients undergoing surgery in center 2 were taken *shortly after the induction of anesthesia* via *arterial catheter*. In contrast, the blood specimens from cancer patients in center 1, as well as control cases from additional sites were exclusively collected *prior to anesthesia by venipuncture* (time: T1). This prompted us to analyze the common effect of anesthesia and procedure of blood withdrawal (venous versus arterial).

**Fig. 2:**
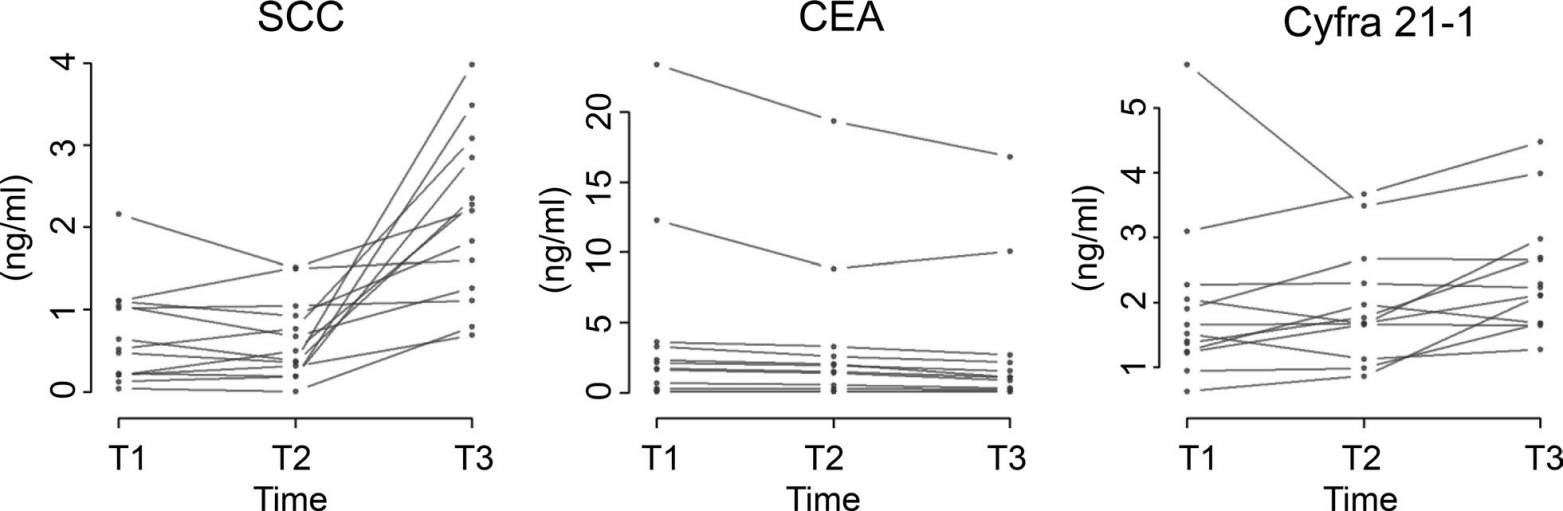
Individual time courses for each marker.

**Fig. 3:**
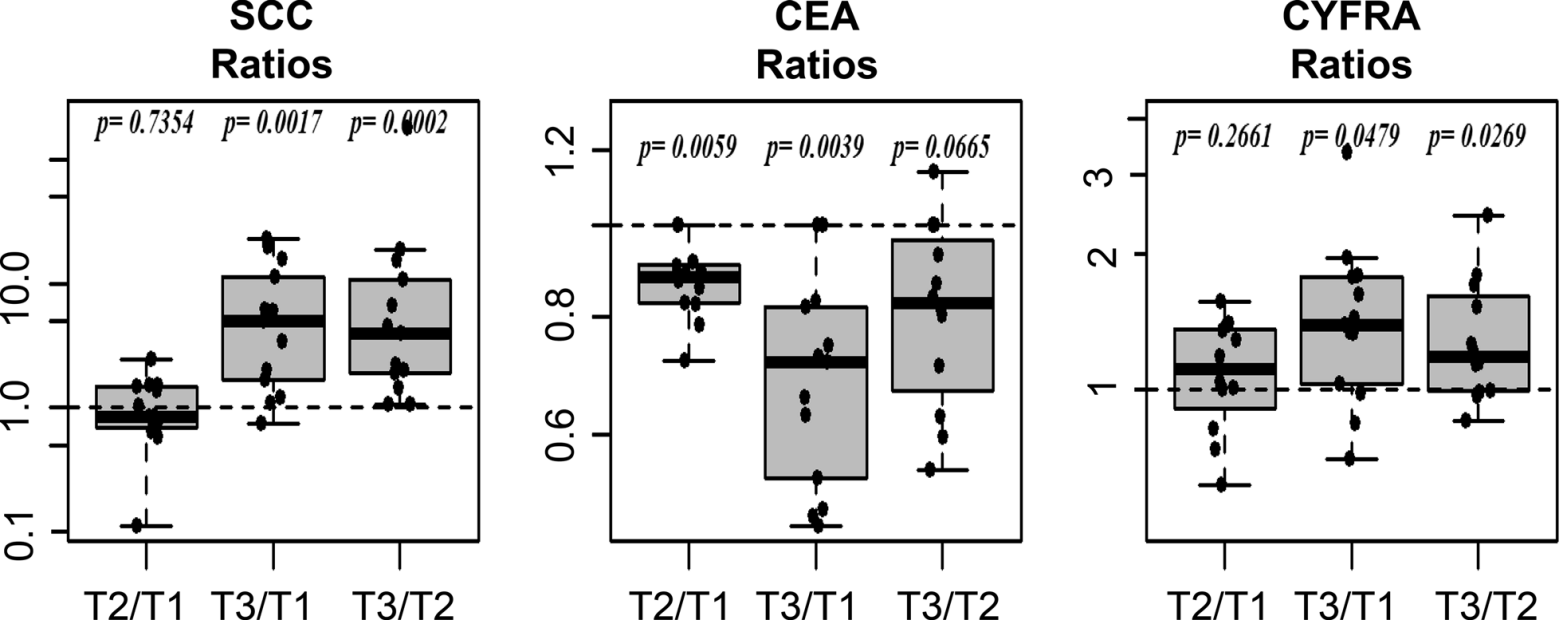
*Boxplot* of ratios of tumor marker levels at points in time T1–T3.

**Fig. 4:**
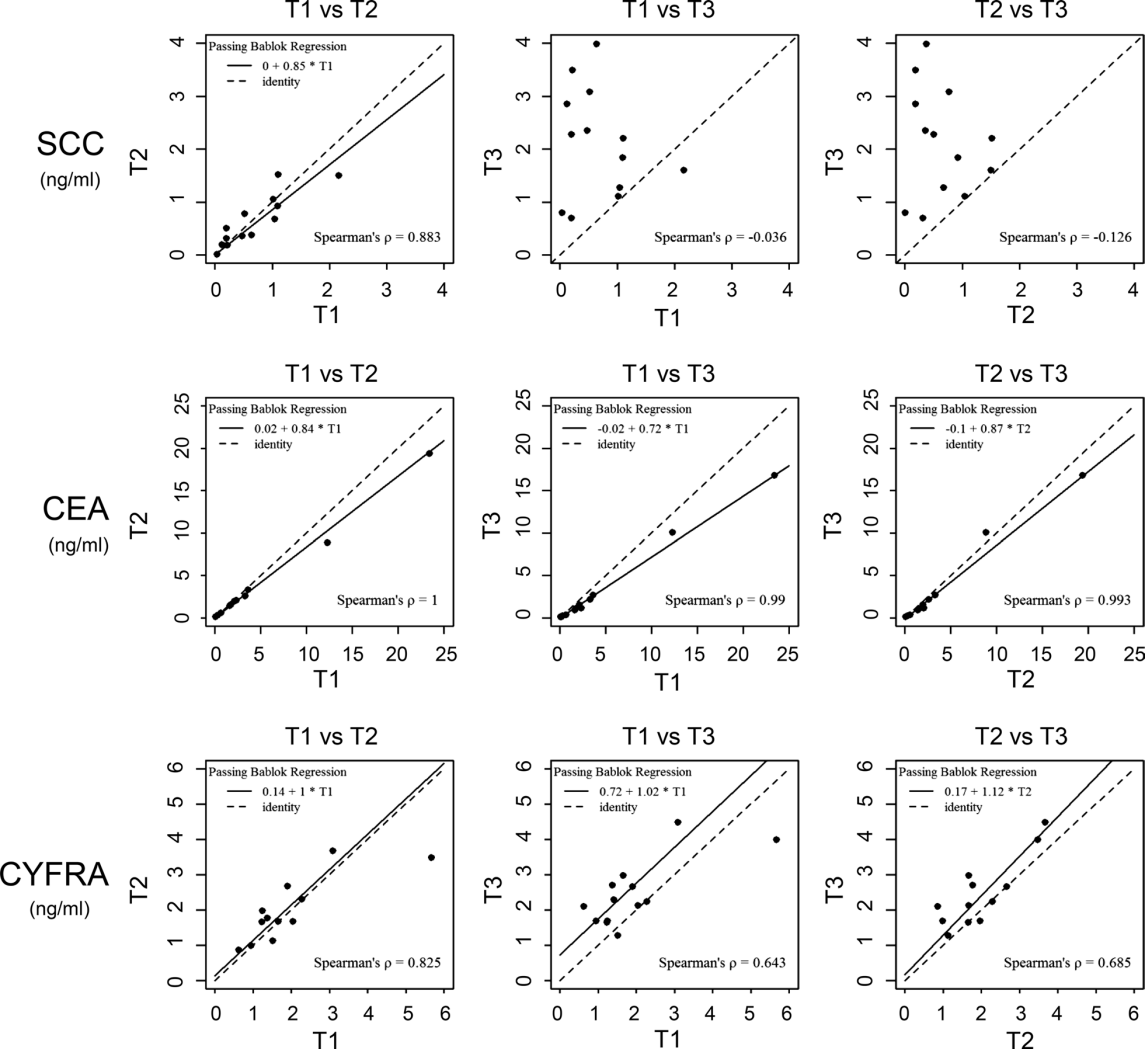
*Scatterplots* of marker concentrations at various points in time. The *plots* show marker concentrations at T1 versus T2, T1 versus T3 and T2 versus T3 for each marker together with fitted regression lines according to Passing Bablok [17].

In Fig. 2 the individual time courses in patients for all three markers are shown. There is a strong increase of the average SCC concentration at time 3 (T3). For CYFRA21-1 a moderate increase of average concentrations at T3 is seen and CEA shows a weak but systematic decrease. Although there might be the impression that the differences in CEA are due to 2 patients with higher CEA values, it is shown in figure that all patients but one had systematically decreased values (Fig. 3). Fold changes (ratios) between individual points in time are summarized in Table 1 and shown in Fig. 3. The fold change for SCC is significantly increased at T3 compared to T1 (p = 0.0017) and T2 (p = 0.0002). The median fold change between time T1 and T3 is most pronounced in SCC (~5 fold) compared to CEA (0.72 fold) and CYFRA 21-1 (1.4 fold). In Fig. 4 the correlation between marker concentrations at T1 and T2, T1 and T3 and T2 and T3 is shown. The correlation between T1 and T2 is fairly good for all markers. On the other hand, there is no correlation between T1 and T3 and T2 and T3 for SCC (ρ = −0.036 and ρ = −0.126, respectively).

**Table 1:** Distribution of marker value ratios

Biomarker	Time ratio	Mean	Median	Min–max	*p* value*
SCC	T2/T1	1.06	0.84	0.11–2.44	0.7354
	T3/T1	7.42	4.96	0.74–22.79	0.0017
	T3/T2	18.84	3.99	1.06–175.67	0.0002
CEA	T2/T1	0.87	0.88	0.72–1.00	0.0059
	T3/T1	0.70	0.72	0.48–1.00	0.0039
	T3/T2	0.82	0.83	0.55–1.14	0.0665
CYFRA 21-1	T2/T1	1.12	1.11	0.61–1.57	0.2661
	T3/T1	1.51	1.40	0.70–3.36	0.0479
	T3/T2	1.34	1.18	0.85–2.43	0.0269

* p value corresponds to the Wilcoxon signed rank sum test for paired data.

A similar but much weaker effect upon the correlation of marker concentrations between points in time can be seen for CYFRA 21-1. There is a decrease in the correlation coefficient from greater 0.8 to less than 0.7. Most data points show an increase in concentration at T3 compared to T1 and T2. However, CEA shows a good correlation (ρ > 0.9) for all time points.

## Discussion

Using standardized and commercially available assays to validate candidate biomarkers has been proven to be feasible and reliable. Biobanks are considered as an important source for rapidly available samples with good clinical characterization. However, many biobanks supplied by multicenter studies suffer from inadequately characterized pre-analytical variables that may have a major impact on marker concentrations detected.

In this regard, there are international efforts to better characterize biospecimens by annotating pre-analytical variables that may have an influence on biomarker levels. Betsou et al. [16] suggested the use of SPREC coding (sample PREanalytical code). However, in the proposal, specific codes for the time point of sampling and for procedures (venipuncture versus arterial sampling vs. capillary sampling) have not been considered so far. The main focus of SPREC was on sample processing and storage conditions. However, our results clearly suggest, that variables prior to sample acquisition should be also annotated.

Patient derived factors of variance such as fasting, exercise and particularly medication, may be expected to show a considerable influence on biomarker levels seen. Tranquillizers taken prior to surgery or endoscopy or drugs used prior to the induction of anesthesia may also affect marker levels seen. Furthermore, it is known that the method of sample collection i.e. venipuncture versus arterial catheter versus capillary puncture may have an influence on the measurement of small molecules (for example platelet–monocyte aggregates) [17]. However, it is difficult to separate the effect of arterial versus venous blood sampling on tumor marker levels without the additional effect of anesthesia. Arterial blood sampling performed in the manner used in our study certainly requires anesthesia. The latter we think is the more important confounder in our setup.

Apart from variability induced by the blood sampling technique used; inter- and intraassay variation and biological variation could contribute to differences in measured values in our study. As all samples were measured within one assay run, interassay variation did not play a role in our study. For CEA, repeatability has been reported by the manufacturer (Roche, Elecsys) as being between 1.3 and 5.0% depending on the amount of analyte in the sample (technical bulletin CEA, Roche). For CYFRA 21-1 the corresponding values were reported as between 1.6 and 2.1% (technical bulletin CYFRA 21-1 Roche). Respective data for SCC were between 1.9 and 2.4% (technical bulletin SCC CanAG Fujirebio). The biological variation of CEA, CYFRA 21-1 and further markers were analysed by Trapé et al. [18]. They did not find significant differences in biological variation for CEA and CYFRA 21-1 between the control group and lung cancer patients.

At least for the tumor markers that we analysed, there are no reports available on circadian differences as a potential source of within-patient variation, for example when a blood sample is withdrawn in the morning, in the afternoon or evening from the same patient To date we do not fully understand the mechanisms by which the concentration alterations were induced. However, it seems to be very unlikely that the changes that we have seen in protein tumor marker levels might be caused by de novo synthesis or degradation of protein within the relatively short time period between induction of anesthesia and blood collection. It is more likely that the changes have been induced by biomarker release from reservoirs in response to the medication, for example from interstitial space, or cell cytoplasm. Small variations or decreasing marker levels, as we described in case of CEA may be attributable to differences in venous versus arterial blood levels. Another possible explanation would be a direct interference of the drug (anesthetics) with the immunoassay. However, the underlying mechanism for this alteration and differences in respect to specific sub cohorts for example gender, age and race remains to be elucidated in further studies. This requires obviously larger patient cohorts and in vitro experiments. However, this was clearly not the scope of the present study.

We acknowledge that our study population is a rather small with some heterogeneity in respect to underlying lung disease. However, the number of patients was adequate to provide significant results in robust Wilcoxon test, and which is known not to be influenced by extreme values.

Furthermore, there is a lack of available literature focusing specifically on systematic pre-analytical research, particularly upon potential variability seen in biomarker levels at different sampling times or before and after specific diagnostic or therapeutic procedures. To date we are aware of only one study that evaluated similar blood sampling conditions in relation to the concentration of established tumor markers (CA 125, prolactin) in ovarian cancer. Thorpe et al. [19] established that the time point of blood sampling (before surgery and after induction of anesthesia) had a direct effect on prolactin levels. Our study shows, that if conditions of the blood sampling are not highly annotated the results seen might eventually lead to misinterpretation of results (for comparison see Fig. 1). However, the biological as well as the clinical relevance of our findings remains to be defined.

In summary, there are two important results from this study—first, biomarker values may be systematically biased if the sample is taken prior to surgery or subsequent to anesthesia in the operation theatre, and secondly, the influence, such as increase, decrease or stable concentration of individual markers may be different for each marker and cannot be predicted.

There are two ways out from this dilemma, first, to check the influence of every potential variable on marker concentration and second to minimize variation between centers by controlling for all currently known preanalytical parameters which might influence the measurement results.

The latter might be accomplished by better annotating pre-analytical variables which might facilitate the selection of samples with common pre-analytical history. One initial step in this direction might be the introduction of SPREC coding. However, SPREC should be supplemented with preprocessing patient derived variables as proposed by this study.

## Conclusions

We conclude that the non-adherence to sampling protocols for biomarker validation studies imposes a significant risk for bias. These results are a caveat for researchers to ensure that all biomarker sampling protocols are controlled for these type of influences.

## Methods

Blood specimens were taken from 13 consecutively recruited patients at different time points prior to surgery. Blood samples were withdrawn 1–2 days prior to surgery by venipuncture (T1) between 8 and 12 a.m., on the day of surgery before anesthesia by venipuncture (T2) and after induction of anesthesia via an arterial catheter (T3). There were 10 male and 3 female patients. The mean age was 59 years. One patient had a hamartoma of the lung, 3 patients underwent resection for lung-metastasis from non-pulmonary primary cancers and 9 patients suffered from non-small cell lung cancer (NSCLC) (Table 2). All samples were taken prior to any surgery. The patients underwent no specific cancer treatment in between the sampling intervals.

**Table 2:** Patient characteristics

Pat. nr.	Age	Gender	Diagnosis
1	83	M	NSCLC
2	55	M	NSCLC
3	22	M	Lung metastasis
4	55	F	NSCLC
5	65	F	NSCLC
6	61	M	NSCLC
7	70	M	NSCLC
8	68	M	NSCLC
9	62	F	NSCLC
10	57	M	NSCLC
11	50	M	Hamartoma
12	59	M	Lung metastasis
13	64	M	Lung metastasis

Sample collection, processing and storage were performed in standardized manner. Briefly, samples were allowed to clot for 60 min and centrifuged for 10 min at 2,000×*g* at 10°C. Supernatants were stored in 1 ml aliquots at −80°C before use. All samples had the same SPREC code (SER-ZZZ-A-D-N-B-D; according SPRECbase v1.0) [16]. All samples were measured as unicates for automated ELECSYS assays and as duplicates in case of SCC in a single run. The following markers were analyzed according to manufacturer’s instructions: Squamous Cell Carcinoma antigen (SCC) (CanAG SCC EIA, Fujirebio Diagnostics, Malvern, USA), CYFRA 21-1 and Carcinoembrionic Antigen (CEA) Roche Diagnostics GmbH, Mannheim, Germany).

### Statistical methods

Statistical analysis was performed using R version 2.13.2 (http://www.R-project.org/.). For each marker candidate the correlation between different blood samplings was assessed by scatterplots together with Spearman’s correlation coefficient ρ and a Passing Bablok linear regression fit [20]. The regression line is only fitted in case there is a correlation between measurement values. Differences between sampling times are expressed in terms of ratios. Ratios correspond to the observed fold changes and allow for a comparison of effect size between marker candidates. Statistical differences between the various sampling time points were assessed using Wilcoxon signed rank test. A p value <0.05 was considered significant.

## Abbreviations

CEA: carcinoembrionic antigen
CYFRA 21-1: cytokeratin fragment 21-1
SCC: squamous cell carcinoma
NSCLC: non-small cell lung cancer
SCLC: small cell lung cancer
COPD: chronic obstructive pulmonary disease
SPREC: sample PREanalytical code
